# Supplementary material for: Role and mechanism of NCAPD3 in promoting malignant behaviors in gastric cancer
Source: Front Pharmacol. 2024 Apr 22;15:1341039. doi: 10.3389/fphar.2024.1341039 (PMC11070777; doi:10.3389/fphar.2024.1341039)
Supplement: Supplementary file 11 [file DataSheet2.ZIP › GSEA/Transcipt factor/my_analysis.Gsea.1599461814734/TGANTCA_AP1_C.html]

Details for gene set TGANTCA\_AP1\_C[GSEA]

|  || Dataset | filtered\_dataset.sample\_info.cls#WT\_versus\_NCAPD3\_MUT |
| Phenotype | sample\_info.cls#WT\_versus\_NCAPD3\_MUT |
| Upregulated in class | NCAPD3\_MUT |
| GeneSet | TGANTCA\_AP1\_C |
| Enrichment Score (ES) | -0.3366312 |
| Normalized Enrichment Score (NES) | -2.5787458 |
| Nominal p-value | 0.0 |
| FDR q-value | 0.0019371766 |
| FWER p-Value | 0.003 |
Table: GSEA Results Summary

  

Fig 1: Enrichment plot: TGANTCA\_AP1\_C      
 Profile of the Running ES Score & Positions of GeneSet Members on the Rank Ordered List

  

| SYMBOL | TITLE | RANK IN GENE LIST | RANK METRIC SCORE | RUNNING ES | CORE ENRICHMENT || 1 | 7763 | ZFAND5 | 26 | 1.006 | -0.0031 | No |
| 2 | 3654 | IRAK1 | 69 | 0.856 | -0.0211 | No |
| 3 | 4204 | MECP2 | 70 | 0.856 | -0.0068 | No |
| 4 | 153769 | SH3RF2 | 82 | 0.824 | -0.0015 | No |
| 5 | 4193 | MDM2 | 131 | 0.769 | -0.0254 | No |
| 6 | 23023 | TMCC1 | 181 | 0.695 | -0.0514 | No |
| 7 | 145389 | SLC38A6 | 202 | 0.673 | -0.0555 | No |
| 8 | 4836 | NMT1 | 208 | 0.669 | -0.0482 | No |
| 9 | 23001 | WDFY3 | 240 | 0.642 | -0.0612 | No |
| 10 | 528 | ATP6V1C1 | 254 | 0.625 | -0.0608 | No |
| 11 | 152485 | ZNF827 | 268 | 0.618 | -0.0604 | No |
| 12 | 5036 | PA2G4 | 365 | 0.557 | -0.1247 | No |
| 13 | 8573 | CASK | 368 | 0.554 | -0.1170 | No |
| 14 | 5718 | PSMD12 | 372 | 0.552 | -0.1101 | No |
| 15 | 9815 | GIT2 | 394 | 0.540 | -0.1171 | No |
| 16 | 23315 | SLC9A8 | 398 | 0.537 | -0.1105 | No |
| 17 | 9743 | ARHGAP32 | 413 | 0.528 | -0.1124 | No |
| 18 | 23242 | COBL | 426 | 0.516 | -0.1130 | No |
| 19 | 3184 | HNRNPD | 508 | 0.470 | -0.1672 | No |
| 20 | 64324 | NSD1 | 534 | 0.455 | -0.1788 | No |
| 21 | 1200 | TPP1 | 536 | 0.454 | -0.1720 | No |
| 22 | 84335 | AKT1S1 | 572 | 0.437 | -0.1915 | No |
| 23 | 219743 | TYSND1 | 607 | 0.419 | -0.2106 | No |
| 24 | 11083 | DIDO1 | 631 | 0.406 | -0.2215 | No |
| 25 | 286410 | ATP11C | 637 | 0.405 | -0.2185 | No |
| 26 | 6197 | RPS6KA3 | 643 | 0.403 | -0.2156 | No |
| 27 | 57679 | ALS2 | 656 | 0.399 | -0.2182 | No |
| 28 | 84085 | FBXO30 | 671 | 0.389 | -0.2224 | No |
| 29 | 6427 | SRSF2 | 679 | 0.388 | -0.2213 | No |
| 30 | 149076 | ZNF362 | 680 | 0.388 | -0.2149 | No |
| 31 | 55122 | AKIRIN2 | 721 | 0.363 | -0.2395 | No |
| 32 | 80218 | NAA50 | 782 | 0.332 | -0.2799 | No |
| 33 | 8904 | CPNE1 | 814 | 0.308 | -0.2985 | No |
| 34 | 7429 | VIL1 | 827 | 0.299 | -0.3027 | No |
| 35 | 6303 | SAT1 | 850 | -0.233 | -0.3157 | No |
| 36 | 3673 | ITGA2 | 858 | -0.264 | -0.3167 | No |
| 37 | 571 | BACH1 | 884 | -0.300 | -0.3308 | Yes |
| 38 | 5292 | PIM1 | 885 | -0.300 | -0.3258 | Yes |
| 39 | 64063 | PRSS22 | 896 | -0.315 | -0.3282 | Yes |
| 40 | 9181 | ARHGEF2 | 902 | -0.326 | -0.3266 | Yes |
| 41 | 4616 | GADD45B | 904 | -0.330 | -0.3218 | Yes |
| 42 | 23710 | GABARAPL1 | 913 | -0.341 | -0.3223 | Yes |
| 43 | 3691 | ITGB4 | 926 | -0.352 | -0.3256 | Yes |
| 44 | 3489 | IGFBP6 | 934 | -0.359 | -0.3250 | Yes |
| 45 | 153830 | RNF145 | 940 | -0.363 | -0.3228 | Yes |
| 46 | 6273 | S100A2 | 942 | -0.365 | -0.3174 | Yes |
| 47 | 4973 | OLR1 | 944 | -0.366 | -0.3121 | Yes |
| 48 | 9572 | NR1D1 | 977 | -0.392 | -0.3301 | Yes |
| 49 | 9180 | OSMR | 981 | -0.394 | -0.3258 | Yes |
| 50 | 23499 | MACF1 | 989 | -0.398 | -0.3246 | Yes |
| 51 | 54985 | HCFC1R1 | 992 | -0.399 | -0.3194 | Yes |
| 52 | 687 | KLF9 | 996 | -0.402 | -0.3150 | Yes |
| 53 | 7277 | TUBA4A | 997 | -0.404 | -0.3083 | Yes |
| 54 | 9586 | CREB5 | 1009 | -0.409 | -0.3099 | Yes |
| 55 | 339665 | SLC35E4 | 1013 | -0.413 | -0.3053 | Yes |
| 56 | 51646 | YPEL5 | 1014 | -0.413 | -0.2984 | Yes |
| 57 | 3914 | LAMB3 | 1015 | -0.414 | -0.2915 | Yes |
| 58 | 3516 | RBPJ | 1021 | -0.419 | -0.2883 | Yes |
| 59 | 51200 | CPA4 | 1035 | -0.434 | -0.2910 | Yes |
| 60 | 54676 | GTPBP2 | 1050 | -0.447 | -0.2943 | Yes |
| 61 | 6016 | RIT1 | 1072 | -0.458 | -0.3028 | Yes |
| 62 | 3918 | LAMC2 | 1088 | -0.466 | -0.3065 | Yes |
| 63 | 5268 | SERPINB5 | 1089 | -0.467 | -0.2987 | Yes |
| 64 | 3669 | ISG20 | 1097 | -0.475 | -0.2961 | Yes |
| 65 | 8727 | CTNNAL1 | 1106 | -0.482 | -0.2942 | Yes |
| 66 | 3963 | LGALS7 | 1107 | -0.483 | -0.2862 | Yes |
| 67 | 2012 | EMP1 | 1119 | -0.494 | -0.2864 | Yes |
| 68 | 92014 | SLC25A51 | 1155 | -0.519 | -0.3045 | Yes |
| 69 | 639 | PRDM1 | 1159 | -0.523 | -0.2981 | Yes |
| 70 | 79056 | PRRG4 | 1164 | -0.532 | -0.2923 | Yes |
| 71 | 2119 | ETV5 | 1176 | -0.545 | -0.2916 | Yes |
| 72 | 6698 | SPRR1A | 1178 | -0.548 | -0.2833 | Yes |
| 73 | 54407 | SLC38A2 | 1182 | -0.553 | -0.2763 | Yes |
| 74 | 2152 | F3 | 1188 | -0.559 | -0.2708 | Yes |
| 75 | 3866 | KRT15 | 1214 | -0.589 | -0.2802 | Yes |
| 76 | 8061 | FOSL1 | 1222 | -0.597 | -0.2756 | Yes |
| 77 | 10221 | TRIB1 | 1229 | -0.604 | -0.2701 | Yes |
| 78 | 7057 | THBS1 | 1242 | -0.620 | -0.2690 | Yes |
| 79 | 1893 | ECM1 | 1250 | -0.637 | -0.2637 | Yes |
| 80 | 11057 | ABHD2 | 1258 | -0.646 | -0.2583 | Yes |
| 81 | 55 | ACPP | 1265 | -0.656 | -0.2520 | Yes |
| 82 | 8796 | SCEL | 1267 | -0.658 | -0.2417 | Yes |
| 83 | 83931 | STK40 | 1281 | -0.669 | -0.2405 | Yes |
| 84 | 861 | RUNX1 | 1293 | -0.687 | -0.2375 | Yes |
| 85 | 5046 | PCSK6 | 1297 | -0.699 | -0.2282 | Yes |
| 86 | 7414 | VCL | 1308 | -0.712 | -0.2239 | Yes |
| 87 | 7031 | TFF1 | 1309 | -0.713 | -0.2121 | Yes |
| 88 | 3892 | KRT86 | 1313 | -0.724 | -0.2023 | Yes |
| 89 | 3084 | NRG1 | 1320 | -0.739 | -0.1946 | Yes |
| 90 | 84803 | GPAT3 | 1327 | -0.748 | -0.1867 | Yes |
| 91 | 1839 | HBEGF | 1328 | -0.751 | -0.1742 | Yes |
| 92 | 5997 | RGS2 | 1336 | -0.770 | -0.1667 | Yes |
| 93 | 1649 | DDIT3 | 1341 | -0.779 | -0.1567 | Yes |
| 94 | 84708 | LNX1 | 1343 | -0.785 | -0.1444 | Yes |
| 95 | 7422 | VEGFA | 1346 | -0.788 | -0.1328 | Yes |
| 96 | 2520 | GAST | 1349 | -0.790 | -0.1212 | Yes |
| 97 | 7706 | TRIM25 | 1357 | -0.817 | -0.1129 | Yes |
| 98 | 255488 | RNF144B | 1362 | -0.834 | -0.1021 | Yes |
| 99 | 3860 | KRT13 | 1367 | -0.847 | -0.0910 | Yes |
| 100 | 6840 | SVIL | 1377 | -0.916 | -0.0826 | Yes |
| 101 | 26298 | EHF | 1378 | -0.919 | -0.0673 | Yes |
| 102 | 5156 | PDGFRA | 1383 | -0.932 | -0.0548 | Yes |
| 103 | 23650 | TRIM29 | 1384 | -0.951 | -0.0389 | Yes |
| 104 | 59277 | NTN4 | 1390 | -0.999 | -0.0261 | Yes |
| 105 | 5055 | SERPINB2 | 1397 | -1.088 | -0.0126 | Yes |
| 106 | 4312 | MMP1 | 1407 | -1.304 | 0.0023 | Yes |
Table: GSEA details [plain text format]

  

Fig 2: TGANTCA\_AP1\_C      
 Blue-Pink O' Gram in the Space of the Analyzed GeneSet

  

Fig 3: TGANTCA\_AP1\_C: Random ES distribution      
 Gene set null distribution of ES for **TGANTCA\_AP1\_C**
